# The Integration of Reference Electrode for ISFET Ion Sensors Using Fluorothiophenol-Treated rGO

**DOI:** 10.3390/bios13010089

**Published:** 2023-01-05

**Authors:** Dae Hoon Kim, Hae Shin Cho, Jin Heung Kim, Da Ae Jo, Hong Gi Oh, Byoung Kuk Jang, Kwang Soup Song

**Affiliations:** 1 Department of Medical IT Convergence Engineering, Kumoh National Institute of Technology, Gumi 39177, Republic of Korea; 2 Center for Nano Bio Development, National NanoFab Center (NNFC), 291 Daehak-ro, Yuseong-gu, Daejeon 34141, Republic of Korea; 3 Department of Research and Development, MCK Tech Co., Ltd., Daejeon 34013, Republic of Korea; 4 Department of Internal Medicine, Keimyung University School of Medicine, Daegu 41931, Republic of Korea

**Keywords:** ion sensor, ion-sensitive field-effect transistors, reference electrode, fluorinated graphene oxide, fluorothiophenol, indium tin oxide

## Abstract

Ion-sensitive field-effect transistors (ISFETs) detect specific ions in solutions that enable straightforward, fast, and inexpensive sensors compared to other benchtop equipment. However, a conventional reference electrode (RE) such as Ag/AgCl is limited on the miniaturization of the sensor. We introduce reduced graphene oxide (rGO), which serves as a new RE, when fluorinated (F-rGO) using fluorothiophenol through the π–π interaction. The circular RE is integrated between a fabricated microscale two-channel ISFET, which is capable of detecting two kinds of ions on an indium tin oxide (ITO) thin-film substrate, using the photolithography process. F-rGO bound to this circular region to function as an RE in the ISFETs sensor, which operated stably in solution and showed a relatively high transconductance (g_m_) value (1.27 mS), low drift characteristic (3.2 mV), and low hysteresis voltage (±0.05 mV). It detected proton (H^+^) ions in a buffer solution with high sensitivity (67.1 mV/pH). We successfully detected Na^+^ (62.1 mV/dec) and K^+^ (57.6 mV/dec) ions in human patient urine using a two-channel ISFET with the F-rGO RE. The F-rGO RE will be a suitable component in the fabrication of low-cost, mass-produced, and disposable ISFETs sensors.

## 1. Introduction

Graphene oxide (GO) is generally produced using the modified Hummer’s method. This chemical process is used to oxidize the graphite and is exploited to produce GO, which contains oxygen functional groups [1]. Removing the surface functional groups of GO or the reduction of GO produces reduced graphene oxide (rGO). rGO has been extensively used in diverse applications as a substitute for pristine graphene because of its relatively high production efficiency, electronic conductivity, large surface area, and thermal stability [2,3]. rGO has a close chemical similarity to pristine graphene, although it has a number of sp^3^ sites [4].

Ion-sensitive field-effect transistors (ISFETs) can be used to detect many kind of ions depending on the ion-selective membrane (ISM) on the gate channel surface [5,6,7,8]. The PVC membrane that makes up the ISM is composed of molecules that react with specific ions. In an ISFET, the ISM is located on the gate surface and reacts with the specific ions that have entered through the membrane and acts as a barrier to prevent other ions from reaching the sensor surface. Only the specific ions functionalized with the membrane can pass through and transfer charge to the gate of the ISFET [6]. These ISFET sensors require a reference electrode (RE) to accurately determine the potential in an electrolyte solution [7,8]. An Ag/AgCl RE has been widely used because it has high reproducibility and stable working characteristics, even in harsh environments [9,10,11,12]. However, Ag/AgCl Res have limitations in terms of miniaturization because the structure requires an Ag wire and a filling solution, which hinders the possibility of integration with ISFETs and leads to high production costs. There is a need for a new RE that can be miniaturized and integrated into the ISFET manufacturing process. We propose a new RE and fabricate an RE-integrated ISFET ion sensor that can stably detect proton (H^+^), sodium (Na^+^), and potassium (K^+^) ions in human urine. Using a radio frequency (RF) sputtering method, we deposited an indium tin oxide (ITO) thin film on silicon substrate and fabricated the uniform ISFETs using a photolithography process. The graphene RE was manufactured using fluorinated rGO (F-rGO RE) treated through the π–π interaction between fluorothiophenol (FTP) and rGO. This F-rGO RE can be manufactured using a semiconductor manufacturing process and produces a miniaturized RE that can be integrated with ISFETs.

## 2. Materials and Methods

### 2.1. Fabrication of Graphene RE and ITO Deposition

The average thickness of the rGO (Graphene Supermarket, New York, NY, USA), which consists of three to eight graphene monolayers, was 3 nm. The rGO was sonicated in an FTP solution for 5 min, heated at 180 °C for 20 min, and cooled at 25 °C. Subsequently, it was centrifuged at 3000 rpm at 25 °C to obtain fluorinated rGO (F-rGO). The resulting F-rGO was dispersed in FTP at 1 mg/mL and then dropped onto the RE surface. The excess unadsorbed F-rGO on the substrate was rinsed off with flowing distilled water. The formed F-rGO layer had an area of 1.77 mm^2^ in contact with the electrolyte.

ITO thin films were deposited on the silicon wafer (Si/SiO_2_) using RF sputtering equipment (Korea Vacuum Tech. KVS-2000L, Korea). The pressure in the chamber was 5 × 10^−6^ Torr, and Ar and O_2_ gas were injected at a ratio of 20:1. Subsequently, the temperature of the substrate was maintained at 100 °C, and the pressure in the chamber was 2 × 10^−2^ Torr. The internal gas was ionized with a power of 200 W, followed by 10 min of pre-sputtering, and thereafter sputtered to deposit the ITO thin film on the Si/SiO_2_ substrate for 210 s. After deposition was completed, the Si/SiO_2_ substrate was held in the chamber for 30 min at 100 °C to ensure the uniformity of the ITO thin film, and then cooled slowly in the chamber in an Ar gas environment.

### 2.2. Ion-Selective Membrane and Buffer Solution

An ion-selective membrane (ISM) for Na^+^ ion detection in solution was made from 2 mg sodium ionophore III and 33 mg PVC, with 65 mg bis(2-ethylhexyl) sebacate as a plasticizer, and 100 mg of this mixture was dissolved in 1 mL tetrahydrofuran (THF). In the ISM for K^+^ ion detection, 2 mg valinomycin and 0.5 mg potassium tetrakis(4-chlorophenyl) borate were added to the lipophilic salt. Then, 32.8 mg PVC and 64.7 mg bis(2-ethylhexyl) sebacate were dissolved in 1 mL THF. The dissolved mixture was dispersed through sonication, and 2 µL was used by drop-casting onto the gate channel surface. Carmody buffer solution (0.2 M boric acid, 0.05 M citric acid and 0.1 M trisodium phosphate) was used as a pH buffer solution adjusted from pH 2 to 12. Ultrapure water (18.2 MΩ⋅cm^−1^) was used. Na^+^ and K^+^ ion solutions were prepared by dissolving NaCl and KCl in a 50 mM Tris-HCl buffer solution. The chemicals were used without any further purification, and pH measurements were performed using a digital pH meter.

### 2.3. Method of Analysis

The crystallinity of the ITO thin films was evaluated using an X-ray diffractometer (XRD, Rigaku SmartLab, Tokyo, Japan). The Cu-α emission line (λ = 1.5418 Å) was scanned in the range of 20° < 2θ < 70°. The thickness of the ITO thin film was measured using a field-emission scanning electron microscope (FE-SEM, JEOL 7610F, JEOL Ltd, Tokyo, Japan). A Fourier-transform infrared microscope (FT-IR, Bruker Hyperion 2000, Bruker, Billerica, Germany) was used to analyze the surface functional groups of the rGO and F-rGO. The properties of rGO and F-rGO were analyzed by Raman spectroscopy (Renishaw 1000, Renishaw PLC, Gloucestershire, UK) using an argon-ion laser at a wavelength of 514 nm.

The electrical properties of the ISFET sensor in the electrolyte solution were measured using a SourceMeter (Keithley 2400, Keithley instruments, Inc., Cleveland, OH, USA) and characterized by the drain–source current (I_DS_), drain–source voltage (V_DS_), and gate–source voltage (V_GS_) in the electrolyte solution. After the electrolyte solution was exchanged, the ISFET sensor was stabilized for 2 min before the steady-state electrical measurements. All electrical measurements were carried out at 25 °C and biased within the potential window of the ITO and F-rGO electrodes to prevent redox reaction.

## 3. Results and Discussion

### 3.1. Evaluation of ITO and F-rGO

**Figure 1:**
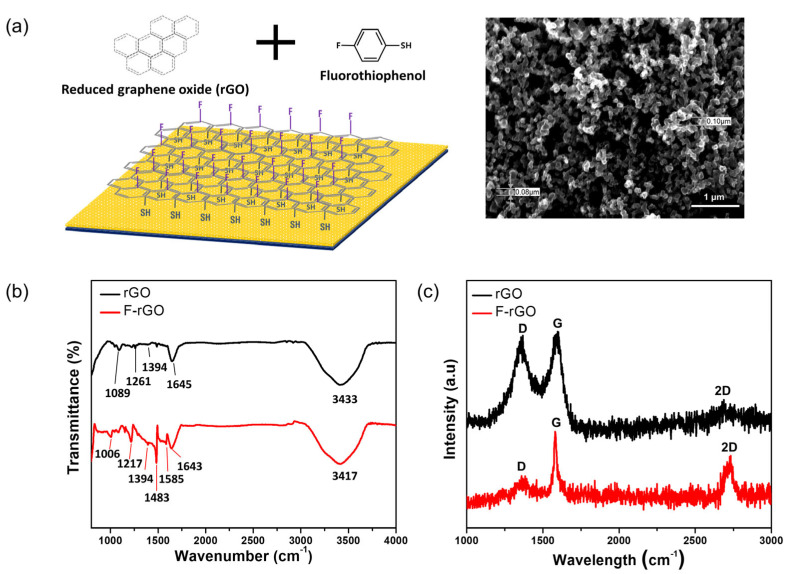
(**a**) Schematic diagram of fluorinated F−rGO and FE-SEM image of the F−rGO on the RE surface. (**b**) FT−IR and (**c**) Raman spectra of the rGO and F−rGO.

The benzene group of FTP is bound to rGO via a π–π bond and functionalizes rGO with fluorine and thiol. The thiol group at the end of the FTP allows the binding of rGO to the Au electrode through thiol bonding (Figure 1a). The F-rGO did not contain solvent residue and was continuous over the entire electrode surface. The rGO exhibited characteristic peaks at 1089, 1261, 1394, 1645, and 3433 cm^−1^ in the FT-IR spectra, as shown in Figure 1b. These peaks were caused by C–O–C bending, C–O stretching, C–OH stretching, the C=C stretching vibration of the unoxidized graphitic domain, and the OH stretching group, respectively. Compared to rGO, F-rGO showed additional characteristic peaks at 1006 and 1217 cm^−1^ that were caused by the C–S stretching vibration and C–F stretching vibration in the benzene ring, respectively [13]. The C=C stretching vibration in the benzene ring was shown at 1483 and 1585 cm^−1^.

The Raman spectra of the rGO and F-rGO are shown in Figure 1c. The D and G bands of the rGO were centered at 1352 and 1596 cm^−1^, respectively, and the intensity ratio (I_D_/I_G_) was 1.42. After fluorination using FTP, the D and G bands were centered at 1349 and 1580 cm^−1^, respectively, and the intensity ratio (I_D_/I_G_) of F-rGO was 0.52. The intensity ratio (I_D_/I_G_) of F-rGO decreased because the disordered rGO that existed on the surface was removed and the oxygen functional groups at the edge states were removed by the FTP treatment. The 2D band increased at 2728 cm^−1^ and the shape of the D band changed after FTP modification. The 2D band is active for crystalline graphitic materials and it is sensitive to the π band in the graphitic electronic structure [14]. The layer of rGO (three to eight graphene monolayers) changed to a light layer (two to five graphene monolayers) after fluorination, and FTP modification was caused by π–π bonding between the benzene ring of FTP and the rGO surface.

Figure S1 is an FE-SEM image of the ITO thin film on the Si/SiO_2_ substrate showing that the film thickness was 35 nm and its crystallinity had a columnar structure. The pattern showed a peak (2θ = 31°) that appeared normal to the (222) plane, indicating ITO thin film, and it demonstrated the typical ITO diffraction pattern, such as (211), (400), (440), and (622) peaks. The peak intensity ratio of the (222) orientation and the (400) orientation peak (I_222_/I_400_) was 1.455 due to insufficient O_2_ flow in the thin-film deposition process [15]. In an environment where the ratio of O_2_/Ar does not exceed 1/13 during deposition, depending on the structure of the chamber, the sheet resistance and lattice constant tended to be lowered [16]. However, we set the O_2_/Ar ratio to 1/20 to increase the resistance of the ITO thin film. As shown previously, high sensitivity to ions has been observed in ITO-ISFET sensors with high gate channel resistance [17].

### 3.2. Characteristics of the ITO-ISFET and F-rGO RE

**Figure 2:**
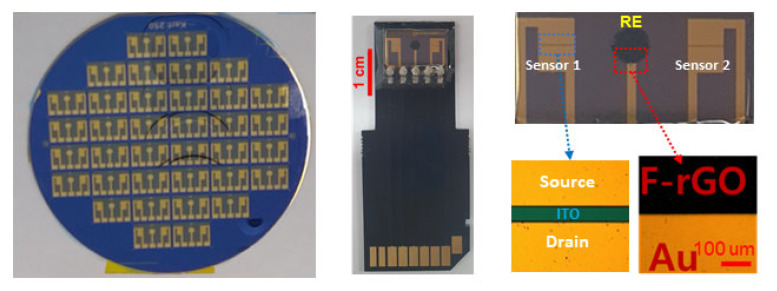
Integrated two-channel ITO-ISFET with F-rGO RE on the sensor tip.

The photolithography process was used to fabricate the drain, source, and gate channel. The RE pattern on the Si/SiO_2_ substrate was fabricated using the same photolithography process, as shown in Figure 2. The gate channel was formed using a photoresist (DNR L300), and ITO was deposited on the Si/SiO_2_ substrate through RF sputtering. After the ITO gate channel was formed on the Si/SiO_2_ substrate, the remaining photoresist was lifted off. The open area of the gate channel in direct contact with the electrolyte solution was 2000 µm in width and 80 µm in length. Patterns of the source and drain electrodes were formed using the DNR L300. Au/Ti was evaporated using a thermal evaporator in a vacuum chamber to make ohmic contact on the source and drain electrodes. An RE with a radius of 0.75 mm was formed by Au/Ti at a location 3 mm away from the gate channel of the ITO-ISFET, and the area of RE in contact with the electrolyte was 1.77 mm^2^. A circular RE electrode was positioned between two ITO-ISFETs at one sensor tip. If the ISM applied to each gate channel is different, two types of ions can be detected simultaneously. It was reported that the single-gate and the double-gate transistors reveal the sub-threshold swings quite close to the theoretical limit at room temperature [18,19]. This means that for a thin channel, there is no necessity to resort to a double-gate structure, which is very inconvenient for fabrication using the semiconductor process.

**Figure 3:**
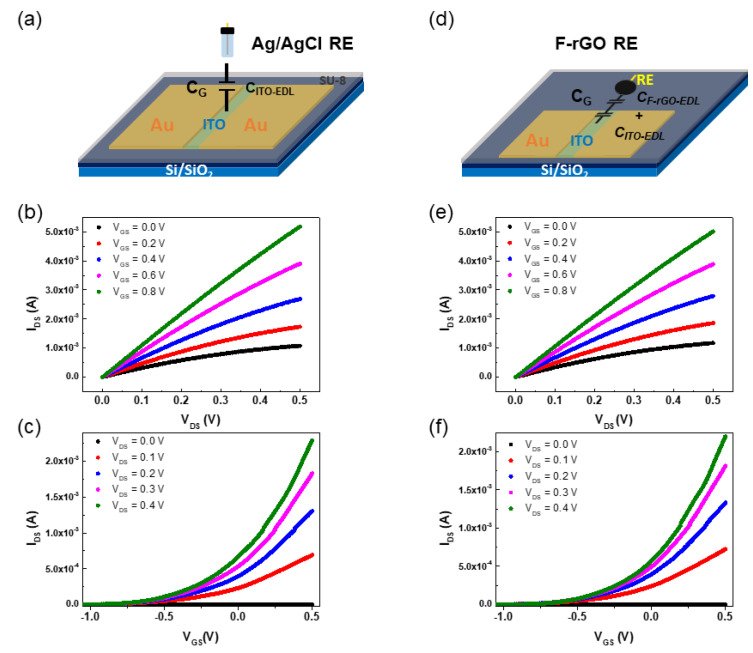
(**a**) Schematic diagram of the Ag/AgCl RE. (**b**) I_DS_−V_DS_ and (**c**) I_DS_−V_GS_ transfer characteristics of ITO−ISFET with the Ag/AgCl RE. (**d**) Schematic diagram of F−rGO RE. (**e**) I_DS_−V_DS_ and (**f**) I_DS_−V_GS_ transfer characteristics of the ITO-ISFET with the F−rGO RE.

The transfer characteristics of the ITO-ISFET with Ag/AgCl and F-rGO REs were evaluated in Carmody buffer solution (CBS) with a fixed pH value (pH 6). Figure 3a shows that the ITO-ISFET with Ag/AgCl RE demonstrated traditional characteristics [20]. The I_DS_ increased depending on the V_GS_ and V_DS_, as shown in Figure 3b. The transconductance (g_m_) of the ITO-ISFET with Ag/AgCl RE was 1.79 mS (V_DS_ = 0.2 V), as shown in Figure 3c.

A schematic diagram of the ITO-ISFET with the F-rGO RE is shown in Figure 3d. As for the sensor structure, a three-dimensional structure (using the Ag/AgCl RE) changed to a two-dimensional structure when the F-rGO RE was used. The I_DS_-V_DS_ and I_DS_-V_GS_ of the ITO-ISFET with the F-rGO RE were the same as those of the general ITO-ISFET with Ag/AgCl, as shown in Figure 3e,f. The g_m_ value of the ITO-ISFET with F-rGO RE was 1.27 mS (V_DS_ = 0.2 V), which was comparable to the g_m_ value when the Ag/AgCl RE was used, as shown in Figure 3f.

When V_GS_ was applied using the F-rGO RE, the ions in the electrolyte moved and formed electrical double layers (<5 nm) on both sides between the gate channel surface of the ITO-ISFET and F-rGO RE. The current flowing through the electrolyte from the ITO-ISFET to the F-rGO RE was negligible (51.2 nA). The electrical double layers had no charge transfer and acted as thin capacitors on both sides. The I_DS_ of the ITO-ISFET is expressed by

(1) $$I_{\mathrm{DS}}=\left(\mu_{n}C_{G}\right)\left(\frac{W}{L}\right)\left[2\left(V_{\mathrm{GS}}-V_{\mathrm{th}}\right)V_{\mathrm{DS}}-V_{\mathrm{DS}}^{2}\right]$$

where μ_n_ is the electron mobility of ITO, C_G_ is the gate capacitance, L is the channel length, and W is the channel width of the ITO-ISFET. The electron mobility of the ITO is 30 cm^2^∙V^−1^∙s^−1^ [21,22]. The C_G_ of the ITO-ISFET (V_DS_ = 0.4 V) with Ag/AgCl RE was 1.16 × 10^−5^ F∙cm^−2^. The C_G_ is the electric double-layer capacitor of the ITO gate channel (C_ITO-EDL_) on the ITO-ISFET with Ag/AgCl RE because the gate channel of the ITO-ISFET was in direct contact with the electrolyte solution. This capacitor acts as a nanogap capacitor at the electrode–electrolyte interface and has high capacitance values (1–100 µF∙cm^−2^) to create a strong electric field at the interface [23]. A high gate capacitance has been pursued to develop high-output currents and to develop biochemical sensors with high sensitivity [24,25]. The C_G_ of the ITO-ISFET with the F-rGO RE was the series connection of C_ITO-EDL_ and the capacitor of F-rGO RE. The capacitor of the F-rGO RE is the electric double-layer capacitor (C_F-rGO-EDL_) because the F-rGO electrode was in direct contact with the electrolyte solution. The C_G_ of the ITO-ISFET (V_DS_ = 0.4 V) with the F-rGO RE was 1.07 × 10^−5^ and the C_F-rGO-EDL_ was 1.42 × 10^−4^ F∙cm^−2^, respectively. The C_F-rGO-EDL_ was high because the F-rGO RE has a large area of overlapping fluorinated graphene nano-electrodes. The value of C_G_ follows the capacitor with small capacitance because the two capacitors connect in series. F-rGO RE has the potential to be used as an RE for ISFETs made of various materials, because the C_F-rGO-EDL_ is larger than the C_EDL_ of other gate channels in the electrolyte.

### 3.3. Ion Detection Using ITO-ISFET and F-rGO RE

**Figure 4:**
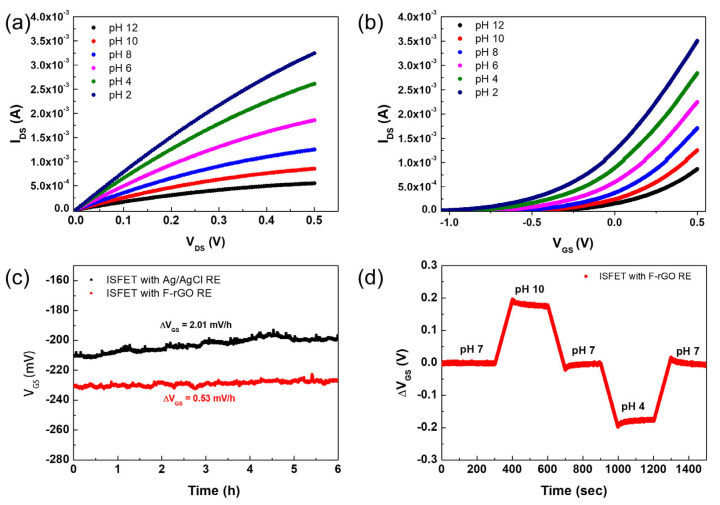
(**a**) I_DS_−V_DS_, (**b**) I_DS_−V_GS_, (**c**) long-term stability, and (**d**) hysteresis characteristics of ITO−ISFET with F−rGO RE in a pH buffer solution.

We fabricated a pH sensor that can detect proton concentrations using a two-dimensional ITO-ISFET sensor based on the F-rGO RE. Figure 4a shows the I_DS_-V_DS_ curve of the ITO-ISFET with the F-rGO RE when the pH value of the buffer solution changes from 2 to 12. The V_GS_ was fixed at 0.25 V, and the V_DS_ was swept from 0.0 V to 0.5 V in each pH buffer solution. The protonation of the gate channel surface of the ITO-ISFET decreases the channel resistance, because ITO is an n-type semiconductor. As a result, I_DS_ increases depending on the proton concentration of the electrolyte. Figure 4b shows the I_DS_-V_GS_ curve of the ITO-ISFET with the F-rGO RE in pH buffer solutions at a fixed V_DS_ (0.5 V). As the pH increased, the V_GS_ of the ITO-ISFET with the F-rGO RE shifted in direction to the right. The ΔV_GS_ was 67.1 mV/pH, which was over the ideal Nernst equation. Similar results were obtained from the ITO-ISFET with the Ag/AgCl RE (ΔV_GS_ was 66.5 mV/pH) (Figure S3 in Supplementary Materials). We evaluated the long-term stability of the ITO-ISFET with F-rGO and Ag/AgCl REs in a buffer solution (pH 6) in real time, which was similar to the drift characteristic of the ISFET [18]. The V_GS_ was continuously measured to keep the I_DS_ at 500 µA with the V_DS_ fixed at 0.5 V on the I_DS_–V_GS_ characteristic, and was continuously maintained at the voltage of −229.0 + 3.2 mV for 360 min, as shown in Figure 4c. The shift voltage of the V_GS_ (0.53 mV/h) was comparable to that of the Ag/AgCl RE (2.01 mV/h) used for 6 h. The hysteresis characteristics of the ITO-ISFET using the F-rGO RE were evaluated. The V_GS_ was continuously measured at fixed values of I_DS_ (500 µA) and V_DS_ (0.5 V) on the I_DS_–V_GS_ characteristics with a periodic injection of CBS at different pH values every 5 min for 25 min, as shown in Figure 4d. The V_DS_ was maintained constant so as to bias the device to work reliably. In the n-channel region, the V_GS_ increased at the high pH to maintain the I_DS_ at a fixed value of V_DS_ on the ITO-ISFET because the surface charge on the gate channel was negative owing to deprotonation at the high pH. In contrast, the surface charge was positive owing to protonation at the low pH, and the V_GS_ decreased to maintain I_DS_ at a fixed value of V_DS_. The hysteresis voltage of the ITO-ISFET using the F-rGO RE was ±0.05 mV at pH 7. The long-term stability and hysteresis voltage of the ITO-ISFET using the F-rGO RE have smaller values than those of the ITO-ISFET using an Ag/AgCl RE [19,26]. The F-rGO RE operates stably in the electrolyte solution and is useful for detecting chemical and biological molecules with high stability.

**Figure 5:**
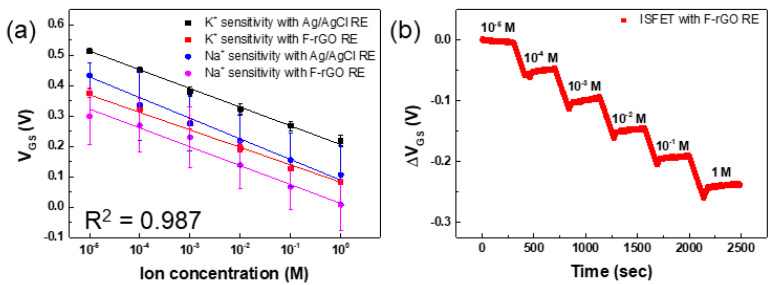
(**a**) Na^+^ and K^+^ ion sensitivities of the ITO−ISFET−ISM with the Ag/AgCl and F−rGO REs. (**b**) Real−time detection of K^+^ ions using the ITO−ISFET−ISM with the F−rGO RE.

We fabricated ion sensors by pasting ISM to the gate channel surface of the ITO-ISFET (ITO-ISFET-ISM) and evaluated the ion sensitivity using Ag/AgCl and F-rGO REs. The stability of the F-rGO was evaluated according to the K^+^ and Na^+^ ion solutions in order to confirm the change of potential according to the ionic strength. The F-rGO had stable potential in the ion solution for each concentration (Figure S6). The sensitivity of the ITO-ISFET-ISM using Ag/AgCl and F-rGO REs in Na^+^ and K^+^ ions dissolved in Tris-HCl buffer solution is shown in Figure 5a. The sensor with sodium ISM exhibited a sensitivity of 62.1 mV/decade and linearity in the Na^+^ ion concentration range of 10^−5^ to 1 M, although the interfering ion (100 mM KCl) was dissolved in the buffer solution. The ΔV_GS_ of the ITO-ISFET with a potassium ISM using the F-rGO RE depended on the K^+^ ion concentration in the Tris-HCl buffer solution. The sensitivity of the K^+^ ion was 57.6 mV/decade, although the interfering ion (100 mM NaCl) was dissolved in the buffer solution. Among various ions contained in the electrolyte, only target ions were detected by the ITO-ISFET-ISM in the Tris-HCl buffer solution. Target ions were captured on the ITO gate channel surface by the ISM. As the concentration of Na^+^ and K^+^ ions increased, the positive charge on the ITO gate channel surface increased; hence, the V_GS_ of the ITO-ISFET-ISM shifted in the negative direction because ITO-ISFET-ISM is an n-type gate channel. We conducted real-time detection of K^+^ ions in Tris-HCl buffer. The V_GS_ was continuously measured with a fixed V_DS_ (0.5 V) and I_DS_ (500 μA) while adjusting the K^+^ ion concentration by periodically injecting a high-concentration KCl solution. The ITO-ISFET-ISM using the F-rGO RE exhibited an immediate and linear response in real time to changes in the K^+^ ion concentration from 10^−5^ to 1 M, as shown in Figure 5b. The ITO-ISFET-ISM using the F-rGO RE has sufficient sensitivity and stability even compared to other ion detection sensors using Ag/AgCl RE, as shown in Table 1. In another study, where the voltage was set as sensitivity using ISFET, Garcia et al. developed a sodium and potassium sensor using a miniaturized Ag/AgCl reference electrode [6]. Fakih et al. and Li et al. confirmed high performance by fabricating the ISFET for potassium detection using graphene [7,8]. Bao et al. developed a 3D-printed hybrid ISFET for sensing ammonium potassium calcium ions [9]. Hu et al. confirmed high sensitivity to Na ions and MB ions through ISM doping of the developed ISFET [10]. In addition, various types of ion sensors have been developed using Ag/AgCl reference electrodes.

In the human body, it is important to monitor concentrations of cations (Na^+^, K^+^, and H^+^ ions), which are found in the blood and extracellular fluid [29] and play an important role in maintaining electrolyte balance, fluid balance, and pH balance in the body [30]. The urine cation test may be necessary after abnormal results have been obtained from a blood cation test, which can identify acute kidney failure [31]. The 24-hour urine cation test can help distinguish between the two common causes of prerenal acute kidney injury (dehydration), and acute tubular necrosis [32]. However, testing using the 24-hour urine analysis is cumbersome and inconvenient. Hence, a robust, easy, and accurate test for monitoring cations is important. We evaluated the ITO-ISFET-ISM using the F-rGO RE on urine samples of patients provided by Keimyung University Dongsan Hospital (IRB No. 2021-08-117). The Na^+^, K^+^, and Cl^-^ ion concentrations in the urine samples are summarized in Table 2. The concentration of each ion in the urine sample was characterized using ion-selective electrode (ISE)-based analytical equipment (ADVIA 2400, Siemens Healthcare, We removed the background color.USA) at Keimyung University Dongsan Hospital.

The sensitivity was simultaneously evaluated by variations in the concentrations of Na^+^ and K^+^ ions in the same urine sample. Figure S4a denotes the I_DS_-V_GS_ of the ITO-ISEFT with sodium ISM using the F-rGO RE in the urine. The V_DS_ was fixed at 0.5 V when V_GS_ was swept from −1.0 to 0.5 V and each concentration was measured five times. The Na^+^ ion concentration in the urine increased from 76 mM to 81, 86, 96, 106, 116, 136, 156, and 176 mM.

**Figure 6:**
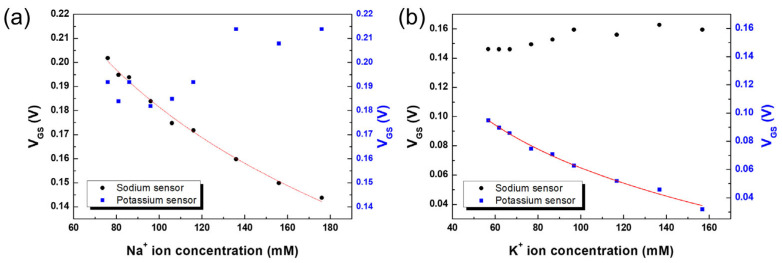
Detection of (**a**) Na^+^, (**b**) K^+^ ions, and the sensitivities of ITO−ISFET−ISM with F−rGO RE sensors in patient urine samples.

The high concentration of Na^+^ ions in the urine sample caused a decrease in the V_GS_ of the ITO-ISEFT with the sodium ISM and shifted the value to the left. Conversely, the I_DS_-V_GS_ of the ITO-ISEFT with the potassium ISM showed irregular shifts with the Na^+^ ion concentration in the urine, as shown in Figure S4b. The sodium sensor in the urine revealed a sensitivity of 69.4 mV/dec, as shown in Figure 6a. Figure S5a shows the I_DS_-V_GS_ of the ITO-ISEFT with the potassium ISM, using the F-rGO RE in the urine at a fixed V_DS_ (0.5 V). The K^+^ ion concentration in the urine increased from 56.8 mM to 61.8, 66.8, 76.8, 86.8, 96.8, 116.8, 136.8, and 156.8 mM; the V_GS_ shifted to the left only for the potassium ISM sensor with the K^+^ ion increment. On the other hand, the I_DS_-V_GS_ of the ITO-ISEFT with the sodium ISM showed irregular shifts with the K^+^ ion concentration, as shown in Figure S5b. The sensitivity of the K^+^ ion sensor was 59.6 mV/dec, as shown in Figure 6b. ISM captured the target ions based on ion size. In the case of the K^+^ ion sensor, since the Na^+^ ion is smaller than the K^+^ ion, it was captured by the potassium ISM on the gate channel surface, resulting in a large interference effect. As in the previous Tris buffer solution as shown in Figure 5b, the detection sensitivity for the K^+^ ions was smaller than that of the Na^+^ ion sensor.

## 4. Conclusions

The rGO was fluorinated using FTP, and the F-rGO was shown to be usable as an RE by sustaining a stable potential despite the change in the solution. The F-rGO RE was integrated into the ITO-ISFET using a semiconductor device fabrication process. These sensors showed the voltage shifts following the Nernstian equation due to changes in the concentrations of cations (K^+^ Na^+^, and H^+^) in the electrolyte solution and showed high linearity. The ISFET with the F-rGO RE has potential as a platform to accurately and quickly diagnose other ions present in human urine.

The imbalance of ions must be carefully managed and accurately diagnosed as some deficiencies (or excesses) may lead to imbalances of other ions. Hospitals demand smart diagnostic equipment to test the pH and anion gap in blood or urine. The ISFET-based ion sensor integrated with the F-rGO RE is one of the best ways to satisfy these requirements. In the present study, although we limited the use of the F-rGO RE to ITO-ISFET sensors, it can also be integrated into silicon, carbon, and other semiconductor-based ISFETs. Therefore, if F-rGO is used as the RE for the ISFETs of various materials, the three-dimensional ISFET type of ion sensor could be converted into a two-dimensional structure and its integration can be broadened, making it possible to develop a low-cost, disposable ion diagnostic sensor.

## Figures and Tables

**Table 1:** Other ion sensors using Ag/AgCl RE.

Reference Electrode	Detection Ion	Sensitivity	Detection Limit	Sensor Type	Ref.
F-rGO	Na^+^ K^+^	62.1 mV/dec 57.6 mV/dec	10 μM	ISFET	This work
Ag/AgCl	K^+^	4.65 uA/uM	0.04 μM	ISFET	[5]
Ag/AgCl	Na^+^ K^+^	62 mV/dec 55 mV/dec	5 mM	ISFET	[6]
Ag/AgCl	K^+^	37 mV/dec	10 nM	ISFET	[7]
Ag/AgCl	K^+^	67 mV/dec	10 μM	ISFET	[8]
Ag/AgCl	NH_4_^+^ K^+^ Ca^2+^	98 mV/dec 104 mV/dec 42 mV/dec	10 μM	ISFET	[9]
Ag/AgCl	Na^+^	60 mV/dec	60 μM	ISFET	[10]
Ag/AgCl	K^+^	53.34 mV/dec	0.06 mM	rGO	[11]
Ag/AgCl	Na^+^K^+^	56.4 mV/dec 54.3 mV/dec	100 μM100 μM	ISE	[12]
Ag/AgCl	Na^+^	56.1 mV/dec	4 μM	ISE	[27]
Ag/AgCl	Na^+^	58.9 mV/dec	42.7 μM	ISE	[28]

**Table 2:** Information regarding patient urine sample.

Urine		
Na^+^ ion (mM)	K^+^ ion (mM)	Cl^−^ ion (mM)
76	56.8	49

## Data Availability

Not applicable.

## Supplementary Materials

The following supporting information can be downloaded at: https://www.mdpi.com/article/10.3390/bios13010089/s1, Figure S1: FE-SEM image of the ITO thin film on Si/SiO_2_ substrate; Figure S2: (a) Schematic diagram of the experiment. pH sensitivity of (b) the pristine graphene ion-sensitive field-effect transistor (PG-ISFET) and (c) fluorinated graphene ion-sensitive field-effect transistor (FG-ISFET) with Ag/AgCl RE in the pH buffer solution; Figure S3: I_DS_-V_GS_ and I_DS_-V_DS_ transfer characteristics of the ITO-ISFET with Ag/AgCl RE in the pH buffer solution; Figure S4: Detection of Na^+^ and K^+^ ions using ITO-ISFET-ISM with F-rGO RE sensors in patient urine samples with varying Na^+^ ion concentrations. The sensitivities of Na^+^ ion sensor (a) and K^+^ ion sensor (b); Figure S5: Detection of Na^+^ and K^+^ ions using ITO-ISFET-ISM with F-rGO RE sensors in patient urine samples with varying K^+^ ion concentrations. The sensitivities of K^+^ ion sensor (a) and Na^+^ ion sensor (b); Figure S6. Stability comparison of Ag/AgCl reference electrode and F-rGO reference electrode in solution A and solution B.
