# Supplementary material for: The Integration of Reference Electrode for ISFET Ion Sensors Using Fluorothiophenol-Treated rGO
Source: Biosensors (Basel). 2023 Jan 5;13(1):89. doi: 10.3390/bios13010089 (PMC9855950; doi:10.3390/bios13010089)
Supplement: Supplementary file 1 [file biosensors-13-00089-s001.zip › biosensors-2127288-supplementary.pdf]

Supporting information

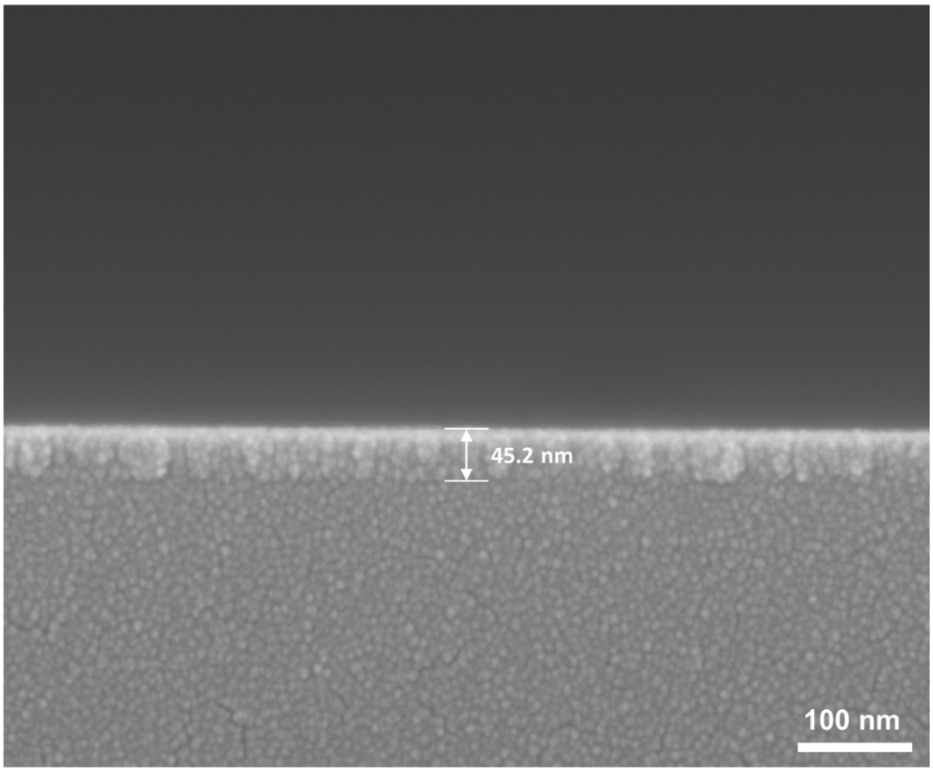

Figure S1. The FE-SEM image of the ITO thin film on Si/SiO_2 substrate.

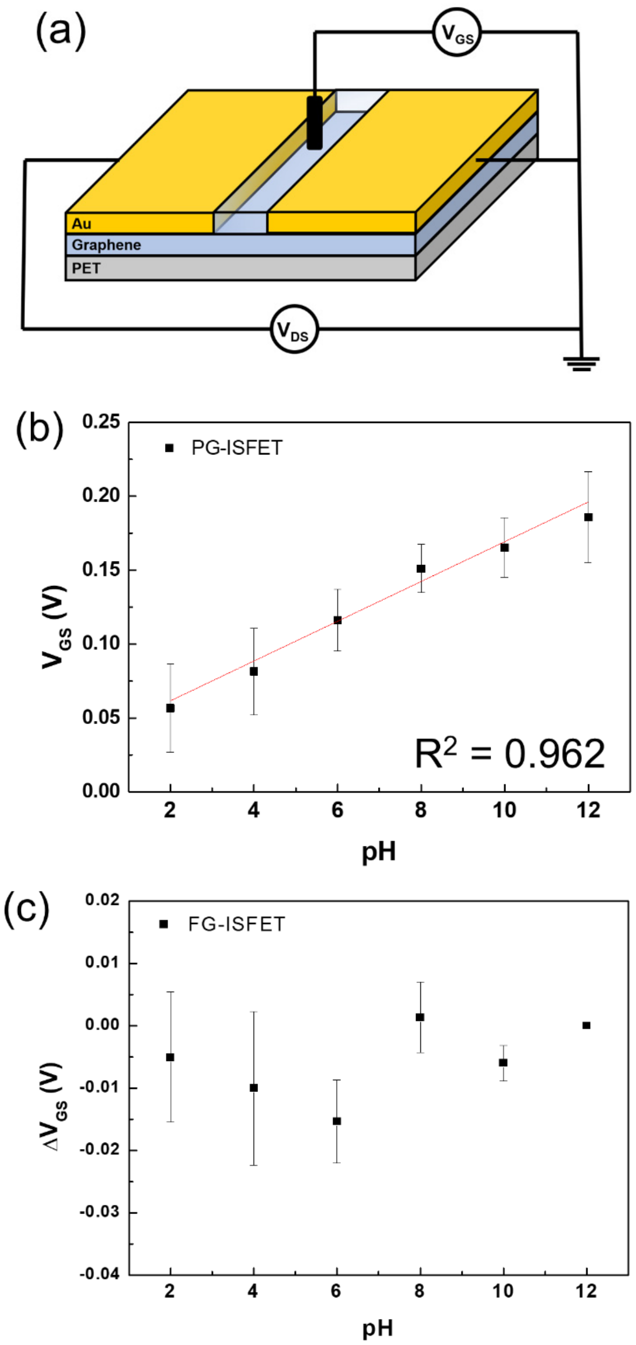

Figure S2. (a) Schematic diagram of the experiment. pH sensitivity of (b) the pristine graphene ion sensitive field-effect transistor (PG-ISFET) and (c) fluorinated graphene ion sensitive field-effect transistor (FG-ISFET) with Ag/AgCl RE in the pH buffer solution.

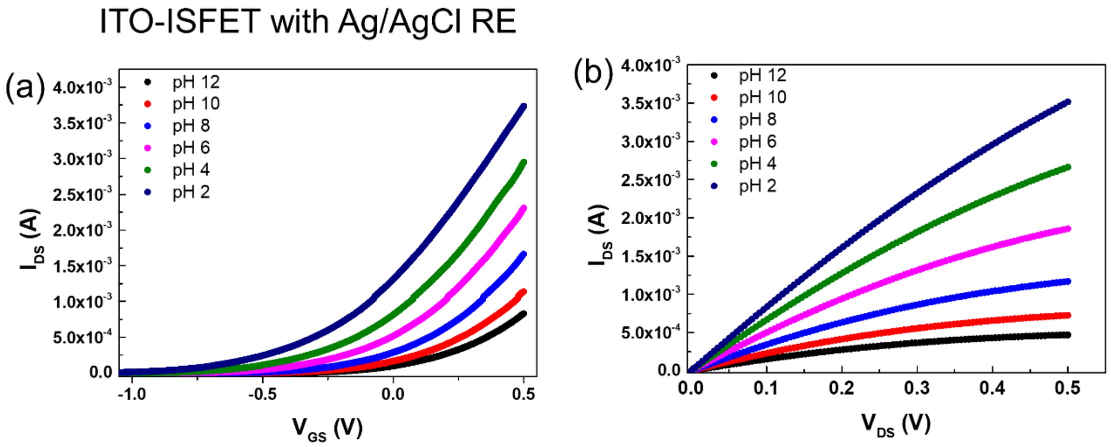

Figure S3. I_{DS}-V_{GS} and I_{DS}-V_{DS} transfer characteristics of the ITO-ISFET with Ag/AgCl RE in the pH buffer solution.

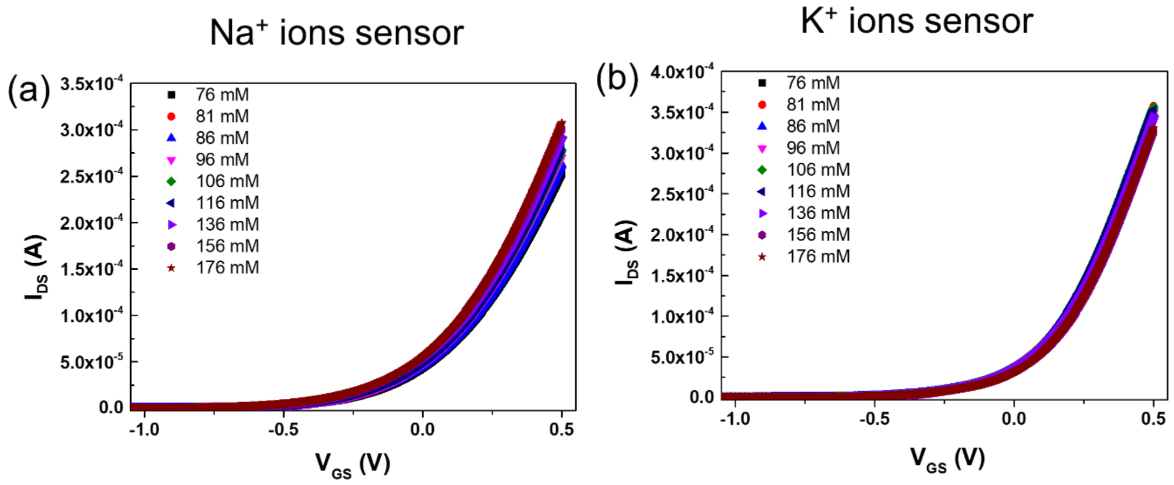

Figure S4. Detection of Na^+, K^+ ions using ITO-ISFET-ISM with F-rGO RE sensors in patient urine samples with varying Na^+ ion concentrations. The sensitivities of Na^+ ion sensor (a) and K^+ ion sensor (b).

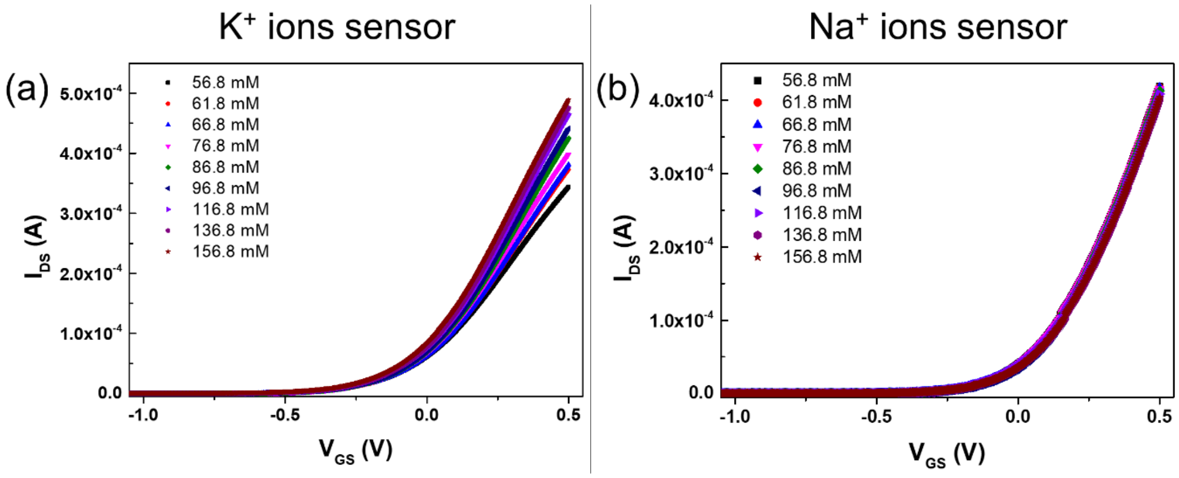

Figure S5. Detection of Na^+, K^+ ions using ITO-ISFET-ISM with F-rGO RE sensors in patient urine samples with varying K^+ ion concentrations. The sensitivities of K^+ ion sensor (a) and Na^+ ion sensor (b).

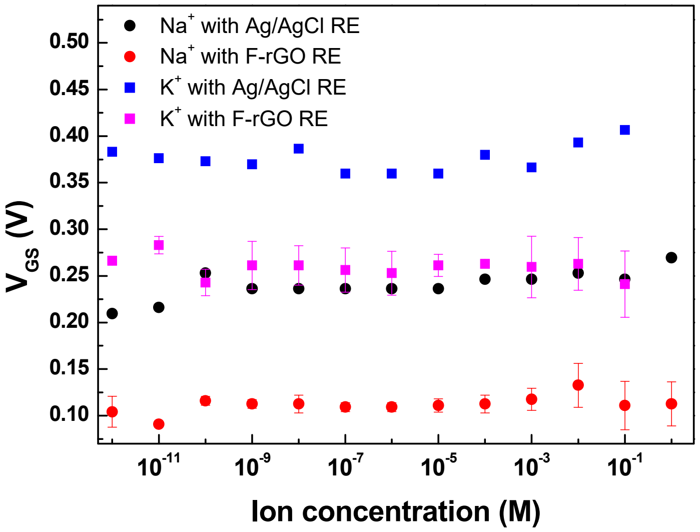

Figure S6. The Stability comparison of Ag/AgCl reference electrode and F-rGO reference electrode in solution A and solution B.
